# Effects of Continuing Oral Risperidone vs. Switching from Risperidone to Risperidone Long-Acting Injection on Cognitive Function in Stable Schizophrenia Patients: A Pilot Study

**DOI:** 10.3389/fpsyt.2018.00074

**Published:** 2018-03-08

**Authors:** Hikaru Hori, Asuka Katsuki, Kiyokazu Atake, Reiji Yoshimura

**Affiliations:** ^1^Department of Psychiatry, University of Occupational and Environmental Health, Kitakyushu, Japan

**Keywords:** schizophrenia, risperidone, risperidone long-acting injection, cognitive function, clinical symptoms

## Abstract

**Objectives:**

Risperidone is the first new generation antipsychotic drug to become available as a long-acting injection (LAI). The purpose of this study was to evaluate the effects of switching from oral risperidone to risperidone LAI (RLAI) on cognitive function in stable schizophrenia patients compared with the effects of continuing oral risperidone.

**Methods:**

Sixteen stable patients who had received risperidone monotherapy for at least 3 months were enrolled (the RLAI group). Before and 24 weeks after switching to RLAI, the Japanese-language version of the Brief Assessment of Cognition in Schizophrenia (BACS-J) and the Positive and Negative Syndrome Scale (PANSS) were administered. To exclude the possibility of learning effects on the BACS-J results, 14 patients with stable schizophrenia who continued oral risperidone treatment were also assessed (the RIS group).

**Results:**

The two groups did not differ with respect to changes in the PANSS score, and no emergent side effects, including extrapyramidal symptoms, were observed. The BACS-J score for verbal memory exhibited greater improvement in the RLAI group than in the RIS group (*p* = 0.047).

**Conclusion:**

The results of this preliminary study suggest that switching from oral risperidone to RLAI may improve verbal capability more than continuing with oral risperidone. However, these findings must be replicated in a larger, double-blind study.

## Introduction

Schizophrenia is a chronic disease with an intermittent course and numerous relapses over time (1). Relapses of schizophrenia are known to adversely affect many biosocial factors (2), and antipsychotic treatment is pivotal for preventing relapse (3). In clinical settings, patients with schizophrenia adherence is often worse and difficult to prevent relapse (4), which greatly impacts the risk of relapse (5).

Risperidone is one of the most widely used atypical antipsychotic drugs for acute schizophrenia treatment. We always continue with maintenance phase treatment after acute phase treatment. However, most patients experience relapse because of low medication adherence, high stress levels, and problems with the use of alcohol and/or drugs. Treatments that are deliverable as long-acting injections (LAIs) are among the more useful therapies in clinical settings.

Recent trends in the treatment of schizophrenia include providing recovery-oriented care, which emphasizes the need to improve cognitive and social functioning such that each patient can achieve his or her treatment goals. Therefore, psychiatrists regard cognitive function as an important treatment target. Recent some meta-analysis shows there were no significant different between LAI antipsychotics and oral antipsychotics regarding efficacy, and safety in schizophrenia (6–9). However, same drug head-to head, but different formulation, studies evaluating the cognitive function of antipsychotics in schizophrenia are lacking.

In this study, we evaluated the effects of switching from oral risperidone to risperidone LAI (RLAI) on cognitive function in stable schizophrenia patients compared with the effects of continuing oral risperidone.

## Materials and Methods

Sixteen patients with schizophrenia who had received risperidone monotherapy for at least 3 months were included in this study (the RLAI group). All patients had been receiving oral or liquid risperidone treatment for at least 3 months. Patients with a concomitant medical state were eligible to participate in the study if their condition had been stable for at least 3 months and they had been receiving standard therapy for the concomitant condition(s) for at least 1 month. Patients were excluded if they had an untreated or unstable clinically significant medical condition, any clinically significant abnormalities upon laboratory examination or physical examination or if they had a thyroid function abnormality. Other reasons for exclusion included a history of seizures, recent drug or alcohol abuse, a principal psychiatric condition other than schizophrenia, and a suicide attempt during the current psychotic episode. To exclude the possibility of learning effects on the cognitive function, 14 patients with stable schizophrenia who took oral risperidone were also evaluated as continuing group (the RIS group).

### Japanese-Language Version of the Brief Assessment of Cognition in Schizophrenia (BACS-J)

Trained psychiatrists assessed cognitive function using the BACS-J (10). The BACS-J, which has well-established reliability and validity, is designed to measure cognitive function in individuals with schizophrenia. Results were adjusted for the influence of age by utilizing age-matched cohorts of controls to calculate BACS-J *z*-scores for each patient with schizophrenia in the present study.

### Procedure

Patients in the RIS group continued their treatment with the same dose of risperidone. This dose had been determined based on each patient’s clinical status, with an upper limit of 12 mg/day. Patients in the RLAI group received an initial 25-mg injection with overlap with oral risperidone for at least 3 weeks. The maintenance target dose for RLAI was 25 mg every 2 weeks, with an allowable dose range of 25–50 mg every 2 weeks based on the clinician’s judgment. After the crossover period, oral supplementation was permitted for acute exacerbations of positive symptoms, but long-term use (>4 weeks) of an ongoing combination of oral antipsychotic and RLAI was not permitted. Injections were given at a treatment room onsite and were typically administered by a nurse practitioner.

Assessments were completed before and 24 weeks after the initial injection by independent raters. Symptoms were assessed using the Positive and Negative Syndrome Scale (PANSS), and cognitive function was assessed using the BACS-J. Daily dose of risperidone were converted to approximate chlorpromazine equivalents (CPZeq) using published article (11). Written informed consent was obtained from all of the participants of this study. The Ethics Committee of the University of Occupational and Environmental Health approved the study protocols, which included standard procedures for clinical research involving vulnerable participants in Japan.

### Statistical Analysis

Only data from patients who completed all 24 weeks of the study were evaluated. The raw data collected at baseline and at the study end point were used for statistical analysis. To ensure group comparability, baseline clinical characteristics were tested using *t*-tests or Pearson’s chi-square test, as appropriate. Repeated-measures analysis of covariance was performed for each cognitive and social variable, with the baseline data serving as the covariate. For the primary analyses, the between-subjects factor was the group (the RLAI and RIS groups), and the within-subjects factor was time (before and 24 weeks after the initial injection). The effects of group, time, and group-by-time (the interaction effect) were examined. All statistical tests were two-tailed, and a *p*-value less than 0.05 was regarded as indicative of significance.

## Results

### Demographic and Clinical Characteristics

Thirty patients were allocated into the two groups at the start of the study. No patients dropped out of the study. Therefore, the final analyses included the 30 patients who completed the study. Demographic data for these patients are shown in Table 1. At baseline, the two groups did not differ significantly with respect to age, onset, sex, total PANSS score, total antipsychotic dose (in CPZeq) or education.

**Table 1:** Demographic and clinical characteristics of the participants at baseline.

	RLAI group	RIS group	*p*-Value
Sex (M/F)	7/9	7/7	n.s.
Age (years)	31.5 ± 5.3	27.8 ± 7.6	n.s.
Education (years)	12.9 ± 2.7	13.6 ± 2.5	n.s.
Smoking	12/4	10/4	n.s.
Onset	24.3 ± 4.6	21.9 ± 3.3	n.s.
PANSS-P	16.5 ± 3.6	17.4 ± 4.5	n.s.
PANSS-N	18.7 ± 2.0	18.1 ± 2.9	n.s.
PANSS-G	32.4 + 6.3	36.0 + 4.8	n.s.
PANSS-T	67.8 ± 7.8	71.4 + 8.6	n.s.
DIEPSS	0.3 ± 0.9	1.2 ± 1.8	n.s.

*PANSS, Positive and Negative Syndrome Scale; P, positive scale score; N, negative scale score; G, general psychopathology subscale score; T, total score; DIEPSS, drug-induced extrapyramidal symptoms scale*.

### Clinical Symptoms and Dosage of Risperidone

Changes in the PANSS score from baseline to the study endpoint did not differ between the two groups (Table 2).

**Table 2:** Cognitive function changes in the RLAI and RIS groups.

	RLAI group			RIS group			Time–group interaction	
			
	0 W	26 W	*p*-Value	0 W	26 W	*p*-Value	*F*	*p*-Value
Chlorpromazine-equivalent of risperidone dosage (mg/day)^a^	278.1 ± 122.3	234.6 ± 64.0	0.015	275.0 ± 152.9	285.7 ± 135.1	0.53	2.58	0.11
PANSS score	67.8 ± 7.8	66.7 ± 7.3	0.049	71.4 ± 8.6	70.3 ± 7.3	0.15	0	0.68
Verbal memory	−1.24 ± 0.91	−0.68 ± 0.72	<0.01	−1.12 ± 1.11	−1.10 ± 0.61	0.92	3.91	0.047
Working memory	−0.98 ± 0.73	−0.84 ± 0.64	0.21	−1.02 ± 0.69	−0.93 ± 0.77	0.49	0.13	0.72
Motor function	−0.82 ± 0.96	−0.69 ± 0.85	0.048	−0.96 ± 0.98	−0.70 ± 0.75	<0.01	0.42	0.52
Verbal fluency	−1.02 ± 0.73	−0.83 ± 0.54	0.048	−1.01 ± 1.32	−0.72 ± 0.88	<0.01	0.56	0.46
Attention and processing speed	−1.60 ± 1.30	−1.21 ± 0.93	<0.01	−1.51 ± 1.13	−1.34 ± 1.00	0.09	1.40	0.25
Executive function	−1.41 ± 2.00	−0.97 ± 1.19	0.02	−1.38 ± 1.96	−0.89 ± 0.95	0.01	0.02	0.89
Composite score	−1.18 ± 0.62	−0.87 ± 0.36	<0.01	−1.17 ± 0.45	−0.95 ± 0.32	<0.01	0.88	0.36

*^a^“Time × group” interaction effect on analysis of variance with BACS-J when compared with the RLAI group*.

At baseline, the RLAI and RIS group did not differ with respect to dose of risperidone. Paired *t*-tests demonstrated that after 24 weeks, dose of the risperidone was significantly decreased in the RLAI group.

### Cognitive Function

At baseline, the RIS and RLAI groups did not differ with respect to any of the BACS *z*-scores. Paired *t*-tests demonstrated that after 24 weeks of treatment, the *z*-scores for verbal memory, motor function, verbal fluency, attention and processing speed and executive function were significantly improved in the RLAI group, and the *z*-scores for working memory, motor function, verbal fluency, and executive function were significantly improved in the RIS group.

In an analysis of changes in BACS *z*-score from baseline to the study endpoint, the verbal memory *z*-score showed greater improvement in the RLAI group than in the RIS group (*p* = 0.047). There were no significant between-group differences in changes in other scores, including the total score for each scale (Table 2).

## Discussion

The most clinically relevant finding obtained in this preliminary study is that patients who switch from oral risperidone to RLAI might demonstrate greater improvement in verbal memory than patients who continue oral risperidone treatment. Although the observed between-group differences in the effects on cognitive function were extremely small, our results might have implications for the treatment of schizophrenia. However, the precise mechanism underlying these results remains unknown. In this study, dose of the risperidone was significantly decreased in the RLAI group. Recently report indicate that negative association was found between verbal memory function and dose of risperidone in schizophrenia (12). Moreover, another study reported that dose reduction of risperidone dosage improve the cognitive function in schizophrenia (13). We speculate that these dose reduction might contribute to significant improvement in RLAI group. Furthermore, our findings may be explained by more stable concentrations of risperidone LAI than of oral risperidone or some oral RIS group participants were low adherence than RLAI group.

In the present study, there were no differences between the two groups with respect to changes in the total PANSS score. This result suggests that continuing oral risperidone and switching from oral risperidone to RLAI exhibit similar efficacy.

This study has several limitations. First, the small sample size increased the risk of false-negative findings. A lack of multiple testing correction may also have resulted in type I errors; however, given the pilot nature of the present study, the results obtained in this investigation should be regarded as preliminary. Therefore, replicate studies are needed, possibly with larger samples and a randomized, double-blind design. Second, this study’s open-label design might have impacted the results, since the expectations of patients or raters might have affected the assessments. Third, we did not consider blood levels of risperidone at the times of cognitive assessments or other assessments. These blood levels may have affected the outcomes of evaluations (14), although drug level fluctuations are considerably smaller for LAIs than for orally administered antipsychotics (15).

In conclusion, this pilot study suggests that relative to continuing oral risperidone treatment, switching from oral risperidone to RLAI may produce greater improvements in verbal memory. However, this finding must be replicated in a larger, double-blind, randomized controlled trial.

## Ethics Statement

Written informed consent was obtained from all of the participants of this study. The Ethics Committee of the University of Occupational and Environmental Health approved the study protocols, which included standard procedures for clinical research involving vulnerable participants in Japan.

## Author Contributions

HH designed the study, performed the cognitive battery, collected the clinical data, performed the statistical analyses, wrote the first draft of the manuscript, and managed the literature searches. RY developed the study protocol and wrote the final manuscript. AK and KA collected the clinical data. All of the authors took part in either drafting the article it critically for important intellectual content, and approved the final manuscript.

## Conflict of Interest Statement

HH has received speaker’s honoraria from Dainippon Sumitomo, Eli Lilly, Janssen, Otsuka, Meiji, and Pfizer. AK has received speaker’s honoraria from Dainippon Sumitomo and Meiji. KA has received speaker’s honoraria from Eli Lilly. RY has received speaker’s honoraria from Eli Lilly, Janssen, Otsuka, Meiji, Mochida, Yoshitomi, and Pfizer.

## Abbreviations

BACS, brief assessment of cognition in schizophrenia; JART, Japanese Adult Reading Test; LAI, long-acting injection; RLAI, risperidone long-acting injection; PANSS, Positive and Negative Syndrome Scale.
